# Purpose in Life of Elite Athletes after Spinal Cord Injury

**DOI:** 10.3390/ijerph18115563

**Published:** 2021-05-23

**Authors:** Agata Goraczko, Grzegorz Zurek, Maciej Lachowicz, Alina Zurek

**Affiliations:** 1 Clinic of Neurorehabilitation, 54-519 Wroclaw, Poland; agagoraczko@gmail.com; 2 Department of Biostructure, University School of Physical Education, 51-612 Wroclaw, Poland; maciej.lachowicz@ideis.pl; 3 Institute of Psychology, University of Wroclaw, 50-137 Wroclaw, Poland; alina.zurek@uwr.edu.pl

**Keywords:** purpose in life, spinal cord injury, athletes, global meaning, qualitative–quantitative study

## Abstract

*Background*: Searching for the meaning of human existence is man’s fundamental orientation. People are free to find meaning in their lives, and while they are not always free to choose the conditions of life, they are free to choose their attitude toward the conditions in which they find themselves. When people experience an unchangeable situation, the most important thing is the attitude they take toward it. This study aimed to identify the sense of meaning in life among elite athletes after a spinal cord injury (SCI) and to analyze the different aspects contributing to this domain. *Methods*: The study involved five athletes with at least national-level achievements in sports prior to a SCI. The study consisted of an interview using a communicator and filling out two online questionnaires—a personal questionnaire and the Purpose in Life Scale. *Results*: Analyzing the quantitative results, four participants achieved results indicating a high sense of meaning in life, while one participant achieved a significantly lower result. *Conclusions*: What affects one’s purpose in life is not so much the objective physical limitation but how much physicality one perceives to have lost as a result of the injury. Elite athletes stay involved in the sporting environment, which prevents the loss of purpose and maintains a sense of meaning at a high level. Both telling the story of your own illness and listening to the stories of others help the process of self-healing.

## 1. Introduction

Viktor Frankl is considered to be the only medical expert to have presented a scientifically and empirically based concept of human’s search for meaning and existence, as well as putting existential questions at the core of his practice [1]. According to Frankl (2006), human beings’ fundamental orientation, taking precedence over all other life projects, is searching for the meaning of human existence, called “the will to meaning” [2]. Meaning can be found in three typical modes, each allowing human beings to transcend themselves: through work and success, through love and the experience of beauty in nature and art, and through suffering. Man is the only species who can show determination to transcend suffering [2]. According to Frankl (1986), “life has meaning up to the very last breath,” and when a person experiences an unchangeable situation, the most important thing is the attitude they take toward it [3]. Indeed, the basic tenet of Frankl’s theory, repeated approvingly in the secondary literature, is that firstly, people are free to find their lives meaningful; secondly, they are not always free to choose the conditions of life; and thirdly, they are free to choose their attitude toward the conditions in which they have found themselves [4,5]. Frankl was the creator of logotherapy, the aim of which, he claimed, is to allow the individual to move beyond limitations and achieve fulfillment [6]. From his experiences in a concentration camp during World War II, he observed that life has meaning under all conditions and that it is psychologically damaging when a person’s search for meaning is blocked [7].

One example of experiencing a difficult, unchangeable situation is an accident leading to damage to the spinal cord, often resulting in chronic physical and mental disorders [8]. Many traumatized people have difficulty coping with disorders and secondary conditions such as infections, chronic pain, persistent fatigue, cognitive disorders, increased mental stress, job loss, and difficulty participating in society. Traumatic life events, such as a spinal cord injury (SCI), affect almost all areas of life and pose a serious threat to the importance that people give to their lives [9]. Such people need to face everyday challenges along with questions on how to live a meaningful life again [9]. For people with a SCI, leaving the hospital and returning to the community, where they must be responsible for their own care, is a particularly stressful and challenging moment [8,10]. During rehabilitation, patients are taught to deal with the physical, mental, and social consequences of living with a SCI. In the process of adaptation and rehabilitation, “global meaning,” which refers to global beliefs and global goals guiding people in their lives, may become a source of direction and continuity [9]. The SCI Adjustment Model (SCIAM) assumes that adaptation depends on a variety of factors: biological and medical (gender, age, injury level), psychological (personality traits, general self-efficacy), social (e.g., social support, family relationships, sexual relations, financial security, access to communities and transport), cultural, political, and religious. A person with an SCI evaluates the situation as negative/irrational or positive depending on the factors that modify it: the balance of positive and negative stressors and the level of personal resources they have at their disposal [8]. The next step is to use the coping strategies available. Predisposing and pre-disease factors have a strong influence on adaptation [11]. According to Catalano (2011), environmental factors and individual differences may pose a risk and cause poor adjustment or, conversely, may be a protective factor that increases the chances of adaptive adjustment [12]. Before optimal adjustment occurs, people need to pass through various linear stages of adjustment, which include denial, anger, bargaining, depression and despair, and finally acceptance of the new reality and a desire to grow following the trauma [13]. Feelings of anger, bitterness, sadness, and depression are not barriers to adjustment unless they persist and become chronic obstacles to long-term acceptance and adjustment [8]. Social support and focus on problem-solving (active and positive coping strategies) increase chances of resilience [8].

Numerous studies have been conducted on the connection between disability, hope, and the sense of life. In a research by Smith and Sparkes, 11 out of 14 former rugby players put their hope solely in recovery, and the pursuit of regaining functional capacity becomes the meaning of their lives [14]. Thomson’s research indicates that purpose in life (PIL) is a powerful predictor of adjustment after SCI, mediating the effects of personality variables and locus control [10]. The Krause et al. study aimed to identify the relationship between personality and purpose in life, and the risk of multiple causes of death after SCI, using data from the SCI Longitudinal Health Study involving 3070 participants. Purpose in life was found to be a protective factor of mortality, especially for pneumonia and influenza. Krause et al. highlight the key role of promoting purpose in life as a means of increasing longevity [15]. The results of the Mota et al. study show that initiatives to promote greater purpose in life can help protect against the development of physical disability among U.S. veterans [16]. According to the research of Leeuwen et al., purpose in life as well as acceptance cognitions, self-efficacy, and mastery show more variability and seem to be particularly promising as targets for interventions, which may lead to an improvement in mental health in people with SCI [17]. Frank also refers to the sense of meaning in life of people with severe diseases. He points out that telling the story of one’s illness helps to understand one’s own suffering, which gives meaning to it and thus influences the process of self-healing. At the same time, the storyteller helps others to understand their suffering [18]. A systematic literature review by Peter et al. shows that a high spirituality and PIL are associated with higher life satisfaction and well-being, better mental health and adjustment, as well as reduced mortality [19]. Peter et al. also indicate that research on the psychological resources in SCI is broad but fragmented, while their relationship with participation has rarely been studied. Therefore, further development of resource-based interventions aimed at strengthening people with SCI is recommended [19].

To our knowledge, no research has been carried out to date concerning the sense of life in professional athletes who have suffered SCI as a result of sport practice. The purpose of this research was to determine the feeling of a sense of life among elite athletes after a SCI and to analyze the different aspects that make up this domain.

## 2. Materials and Methods

### 2.1. Participants

The following criteria were adopted for inclusion in the project: having sporting achievements minimally at the national level (winning a medal in at least one sporting event of national rank) and suffering a SCI while practicing sport. The invitation to participate in the survey was sent by e-mail to 30 athletes from various countries (USA, UK, Austria, Germany, Poland, Brazil, Australia, and India), meeting the above criteria. A positive answer was obtained from five people who gave an interview and filled out an online form containing a personal questionnaire and the Purpose in Life Scale.

The first respondent (R1) was a three-time world champion in BMX (an acronym for bicycle motocross) dirt jumps. He suffered a spinal cord injury during the BMX Dirt Finals as he performed his original double backflip trick. Head impact against the ground caused a C3/4 (cervical) damage and quadrupedal paralysis. Due to the extent of the damage and functional limitations of R1, it is not possible to take up a sporting activity. The second respondent (R2) was twice the junior world champion in ski jumping. During training, mid-flight, his foot slipped out of the ski boot and uncontrolled landing caused a fall and an SCI at the C6/7 level. R2 currently practices rugby in the wheelchair and skiing. The third respondent (R3) was a European karate champion. She suffered an injury to the spinal cord in the thoracic section (Th11/12) at a sports camp resulting from a fall of several meters. R3 currently practices dance in the wheelchair. The fourth respondent (R4) participated in multiple BMX races. His greatest achievement was winning the first place in the Dual Slalom off-road national competition. While riding in mountainous terrain, he suffered an accident that caused his core to be damaged at the C6/7 level. R4 does not currently practice any sporting activity despite possessing functional capabilities to do so. The fifth respondent (R5) has been involved in motorsport all his life. He had been involved in motocross racing for 8 years, with several wins and podiums along the way. His career in this discipline ended with an accident leading to a core injury at Th6. Due to his functional upper limbs, he obtained a driving license and started to practice race car driving and hand-cycling, with national and international success.

### 2.2. Methods

The research consisted of two parts: an interview and filling out an online form containing a personal questionnaire and the Purpose in Life Scale (PILS).

#### 2.2.1. Personal Questionnaire (PQ)

The PQ contained basic sociodemographic questions, as well as those concerning the discipline practiced, the greatest sporting achievements, the level of spinal cord injury, involvement in sport after the accident, medications currently being taken, rehabilitation, and pain and respiratory disorders.

#### 2.2.2. Purpose in Life Scale 

The feeling of meaning of life has been examined using the PILS by Crumbaugh and Maholic, which assesses the extent to which people see their lives as purposeful and meaningful [20,21,22]. The test includes 20 questions, which are answered using a 7-point Likert scale, with 1 reflecting extreme feelings of no purpose, and 7—feelings of strong purpose in life [10]. The questions of the PILS are presented in Table 1. Adaptations of the national scales were used.

#### 2.2.3. Interviews and Analysis

Semi-structured interviews were conducted by the first author using an internet communicator with the option of recording. The interviews lasting 1.5–2 h consisted of a series of questions that were partly targeted and open-ended. The interviews were recorded and then literally transcribed. The transcriptions were analyzed using the inductive thematic analysis method by A.G. and A.Z.: the former has many years’ experience working with people suffering from SCI, and the latter is an experienced clinical psychologist, using qualitative methods in her work. This exploratory approach was best suited to explain the nature of the conceptualized experience of the subjects. There is a growing body of literature exploring a wide range of health issues using inductive thematic analysis, which is considered a useful method of examining various perspectives and observations of unrepresentative groups [23]. The thematic analysis consisted of the following stages: (1) getting acquainted with the data through repeated open reading; (2) searching for content/designation units of a lower order, (3) ordering them into higher-order categories, and then (4) using topics to describe the results obtained from the interviews [23,24]. To identify potential topics, relevant and meaningful interview excerpts were collected first, which were considered as data. The data were systematically verified to provide the name of each identified meaning unit and then placed in a higher-order category. Often the same unit of the meaning of text could be placed in more than one category. All differences in identifying the basic content units, categories, and themes have been resolved through discussions between authors. In this way, the rigor and reliability of recruitment, ordering, and data analysis were ensured and the saturation process was observed [25]. This led to the extension of quantitative data obtained through the PILS to the subjective world of subjects’ experiences, feelings, and thoughts. The quantitative and qualitative research projects allowed our team to reach the unique, subjective life perspectives of the SCI athletes surveyed. The final list of topics included the following: self-certification, value of life, and attitude toward death; new life goals; old and new meanings of life; affirmation of life; and perspective of sport in the new life.

### 2.3. Ethical Aspects

All participants in the study gave their verbal consent to participate in the study, to publish its results, and to be potentially deciphered. The study was approved by the Senate Research Ethics Committee of the University School of Physical Education in Wroclaw, Poland (corresponding ethical approval code: 37/2018, art.27, Dz.U.1997, poz.553).

## 3. Results

### 3.1. Personal Questionnaire

Sociodemographic data of the participants, their age, and the level and age of the SCI are presented in Table 2. The respondents come from four countries (two continents: USA and Europe).

### 3.2. Purpose in Life Scale

The possible score in the PILS ranges between 20 and 140 points. Higher scores suggest a higher perception of meaning in life [26]. Table 3 presents the results of respondents. Respondents 1, 2, 3, and 5 had a high sense of meaning in life, while R4 had a low sense.

### 3.3. Analysis of the Interviews

The thematic analysis of the interviews revealed five topics. They were called the ways of discussion among our team members.

#### 3.3.1. Adaptation to SCI

Serious damage to the spinal cord forces the self-verification of one’s own image and changes the way we think about ourselves, life, and death. In R5 we can see a certain transcendent/timeless affirmative attitude to life, while in R4 we can see the evolution of thinking about life, from the desire to be deprived of life to finding the value of life as such, even in suffering and disability. Often acquiring a disability forces such verification, changes the thinking about life, and creates the need to think differently about the value of life. Even without fitness, life has a value.

R5: “I stopped breathing for some time […] actually, it changed my attitude toward life. […] When I opened my eyes, I felt so lucky to be alive. I did not exactly know what I went through but I had a vague idea because I felt such a terrific pain. It was the attitude which helped me want to recover, to move on, of course. I set the horizon that I wanted to chase and to follow in my life but it was a daily attitude that I wanted to get something to move forward. It did not matter if it was a small or big thing. I was told that I will be paralyzed, it was like ‘well, ok,’ shocking a bit. The range of opportunities for disabled people after this is huge.”

R4: “I’m trying to rebuild my life. I’m not sure what it means right now. I struggled with depression during this time. From the very beginning, I wished they would have just put me to sleep. At first, it was the pain, I just couldn’t take the pain. I wished they would just take me behind the hospital and shoot me like a horse. I just didn’t want to suffer like that anymore. […] I still have a lot of physical pain but the destruction of my life is what is a large part of my depression right now. With this injury, my life just turned over like a basket. I lost my career, lost my retirement, along with my career […]. I’m starting to think about what I could do with my life. You are encouraging me, there is something there. This injury destroyed my self-worth and my self-esteem. My whole value as a person has been at least shaken, if not crushed. I’m to rebuild this whole era of confidence and things.”

#### 3.3.2. New Life Goals

All participants wondered how their lives changed after the accident and during the treatment, and integrated their experiences, which resulted in a new identity after the SCI. They set themselves short- and long-term health, family, and social goals that were inspiring and motivating for them and changed the perspective of the experienced events.

R1: “After some time I was able to keep myself standing, it was so good for my mental condition, it was so inspiring and motivating.”

R2: “I was thinking the whole time the more I’m able to do, the more I have to call myself lucky, although I’m paralyzed from that day on.”

R3: “And if I do have some specific objectives, I do not know, to settle down after graduation. To start a family, and I think these are the goals of life, just like any other person’s. Above all, it is about being happy and not making your health worse. I was thinking about taking up ballroom dancing professionally.”

Individual health objectives are integrated with social ones. Often the “murderous” work on the maximum improvement of one’s own functional capabilities is accompanied by a motivating thought of sharing one’s experience with others in a similar situation. Living with a new human/sportsman identity with the experience of SCI, further social activity, is a life concept and mission to improve the quality of life and motivate other disabled people to go on living. The respondents pointed out that having a passion despite traumatic experiences and their consequences attracts other people and gives them hope.

R1: “I think I have done everything for myself already, so it is time to think about other people. That is what keeps me going. My goal is to make my talk, to adjust to different people, to be able to adjust my talks whoever I am speaking to, business people, children, whoever. My goals are to get me to ride a motorbike. […] If you are passionate about something and you do it someone will follow. It is what happened to me with helping others. I’m passionate so they follow me to help others.”

R2: “In my situation now, my goal is to improve my walking on crutches, or without crutches. […] I feel that there is still much possible. I also want to raise the focus of the public when they build things to think about the disabled, but this is [a] long-term project.”

Some participants emphasized that they are in the process of either seeking or renewing their life goals, and others that they have already found them. A serious obstacle for R4, even a psychological barrier to achieving new life goals, is the lowered, depressive mood he experienced both before the accident and now.

R4: “I’ve dealt with depression a lot throughout my adult life. I had it before. Just got so, so much worse since my injury, with my injury it has been consuming. […] I started breaking it in a couple last months, so maybe there is hope […] The depression and some of the negative feelings that I have been describing have slowed down my socialization. It’s just in a couple of last months that I have become more outgoing and more communicative, even to friends and family. […] I didn’t think I would have a future, so why rekindle a relationship if there is no future, it’s still in evolution, that’s for sure […] My goals are somehow to resume some type of volunteering. The reason I got into that career was that I wanted to help people. I hope that I can find something still that can do that. I was thinking that with that nutritional aspect, maybe I will be able to help even more people than before.”

R5: “I am always on the lookout for new goals. So, at the moment my big goal is to compete the best I can, with my disability, in racing. That is my biggest goal, to make the most of this opportunity.”

#### 3.3.3. Old and New Meaning of Life

The search for, discovery of, and having meaning in life is proving to be the key driving force behind life with a disability. Love and relationships with the loved ones and acting for others define the discovered purpose. Acting for the benefit of other people becomes a life mission for SCI athletes. […] Entering into the perspective of another person, often more difficult than one’s own, frees one from the egocentric existence in the circle of one’s own thoughts about oneself and experiencing one’s own suffering.

R2: “You know it is important for me to do some things, because when you do some things you do not get into the struggle of thinking about the whole situation. Of course, there are some days when this is normal, and this is OK. When you think about it all the time, you have to really watch out not to get into this struggle which pulls you down all the time. Having some things to do prevents that.”

R1: “After my accident, I wore a shirt saying ‘Stay Strong’ on the front and my name on the back. […] It was such a strong message for other people. A clothing company reached out to me and spread it everywhere. It was great. […] that gave me a good feeling and I started doing more […] There is a bad, little skate park. They should do something to get those kids out of shoplifting, dealing drugs, etc. The skatepark is unusable, it is badly built. If I would do that, with the skate park, I would feel amazing. For these kids, it is the last chance. That makes me feel good, makes me feel a purpose.”

Some participants particularly emphasized the importance of family and relations with the loved ones in finding meaning in life, and efforts to fight depression and hopelessness. Love for children and the loved ones, and the awareness of love and concern for those closest to them, have become a discovery, independent of the unfavorable circumstances, of the meaning of life even with a disability.

R1: “My kids, yeah. That’s the main thing that keeps me going. You have to do what you have to do.”

R4: “My motivation is the people that I loved and who supported me through this. I don’t want to let them down. That’s the reason why I think I’m still alive right now. How can I let them down? So, it starts with the closest to me, my wife and my family, even people from my past, going back to the BMX community.”

R5: “I do not remember much, but the constant thing was that my family and friends were there to support me and that was key. There was a time, there was a well couple of European championships where all of my friends would turn up to the hospital and would watch it all with me. They were always there for me, my family was always there for me.”

In opposition to the above statements, there is the reflection of R4, who is on a journey, searching and discovering the meaning of life after the accident. In this process, the patient’s condition is not insignificant.

R4: “It opened my mind that there are still some areas that I can use my knowledge and experience in education. […] I’m doing this volunteering and trying to just stay involved in groups and organizations. You never know who you may come across and what door it may open. […] I’m just getting at this closed path that says that I can’t do anything, I have no value, I have no worth, I’m overcoming that barrier to open my eyes to the possibilities of it. What I need to do soon is to learn to pursue these possibilities.”

#### 3.3.4. The Affirmation of Life as Such

Despite a serious injury and a completely changed life, the study participants appreciate life and find joy. They see the possibilities they still have before them. As celebrities, they realize that their activities are followed by their fans and their attitude can give hope to others, as well as shaping their approach to the difficult situations they experience.

R2: “I just try to make people smile because I know this is great for me too. I’m so happy I can be a person who people are proud of. I think I’m really privileged, I’m so happy I can be today a person who spreads some good news from time to time.”

R5: “I feel I am a very lucky person because of what I have. Because I was able to turn my passion into my profession. This is a great privilege, believe me. I wanted to get my driving license, finish college, get to the university, tick things off the list. So I was ticking and thinking about what will be next […] I’ve been incredibly lucky to have had the opportunities that I had, but there is a very prominent saying at the end of the book, ‘with a positive attitude positive things can happen to you,’ with a negative attitude you are not going to achieve anything. It is like in *Shawshank Redemption*, with a good attitude you can achieve anything you want.”

Participant R4 begins to recognize and appreciate the functional capabilities one has, despite high spinal cord injury, while R5 highlights the fact that many people are not aware of the possibilities they could use.

R4: “I wish it were more so, it’s hard for me to appreciate it more. But I start to embrace it and appreciate the fact that I can stand it, which minimizes the many secondary consequences of the injury.”

R5: “People just do not know about stuff. They are not depressed or something, they just do not know about the opportunities around them. Over the years I came across people, I met them for the first time. And we have a conversation, like, how they got injured etc. What do they do now? And when I say that I am a racing driver and they are really surprised. There is a stigma that disabled people cannot do a lot of stuff. I am a driver, and I am helping them, too. That makes people’s eyes go big. The way I see the world is different but everything is possible. When there is no lift you just need to get down the stairs, it is possible, the process is quite long but it is possible.”

#### 3.3.5. Meaning of Sport in Life

Sport in life before the accident was the basis for character formation and is now the strength of such formed character to live with a disability. The importance of sport in the new life after an accident is not diminishing but is different. R1 gives motivational speeches to various groups of athletes and supervises his sons’ BMX bike training. R3 has a strong connection with the sporting environment, which prevents the loss of one’s sporting identity and makes it easier to find oneself in a new life situation.

R1: “I started doing more talks. I went to the Liverpool football club for a couple of weeks. They had this lady I knew from racing when I grew up. She is in charge in the premier league so I get to go there and get along with many people.”

R3: “Karate before the accident taught me perseverance and diligence, which later became very useful in this daily fight. And last year I missed sport very much. I wanted to train something but I didn’t know what exactly. I talked to people from the university sports association to set up a section for people with disabilities, and this year they succeeded, because they set up an integration section precisely for people with disabilities. One such section is the disabled dance section to which I belong. I have been learning how to dance starting this year, and recently I managed to show it in front of a larger audience, because I danced at a medical university beauty contest.”

Another participant sees the sport he practiced before the accident as the way to shape his character, including the qualities of perseverance and finding the purpose and meaning of life. It is the sport that gives identity and personality to the athlete.

R4: “I have gravitated towards sport, it has done a lot for me, it helped build a lot of my character, my drive, even my ability to go to school, it taught me how to set goals, how to go towards them, and to go beyond these goals to higher goals, those are things that I picked up. I think sport has helped me through life. When I was younger, I came from an area where I could have been involved in problems, drugs, gangs, and things of that nature. Sport always drew me somewhere else, I felt that’s what sport can do. Now it’s different, I don’t need it to keep me out of trouble so to speak but it gives you some more passions in life that you never experienced. Those passions, those good feelings… life would be much harder without them.”

## 4. Discussion

The aim of our study was to explore purpose in life and the factors that form this domain, a concept not studied among elite athletes after a SCI.

Analyzing the quantitative results of the purpose in life test, four participants achieved results indicating a high sense of meaning in life (PILS between 106 and 135 points), and one participant significantly lower (PILS = 67). In the available literature, the highest results (*M* = 111) were achieved by Danish patients of rehabilitation centers in the Leeuwen et al. study, and the result remained stable over time (studies repeated after 6 months and 1 year) [17]. In the Krause et al. study, with 309 participants with traumatic SCI, the median result of the PILS was 100 for men and 93.8 for women [27]. In the Rossini study, with 79 veterans diagnosed with SCI, who were receiving medical treatment at a large midwestern VA medical center, the median of the PILS result was 98.9. The researchers found that sense of life was not correlated with the severity of the participants’ medical injury. However, it was significantly and negatively related to a perceived loss of physical functioning [28]. In our study, R4, despite a better functional condition than the others, felt its disability and limitations more significantly than the others due to severe pain and depressive personality. This indicates that not an objective physical limitation but how much physicality one perceives to have lost as a result of the injury affects purpose in life and each person decides what makes his or her life meaningful. Most respondents scored higher (the average PILS result for R1–R5 was 109.2 and, excluding R4, 120) than in similar studies available in the literature. As an outlier, we can consider the R4 score, which could be caused by the following factors: depressive personality prior to the accident; lack of involvement in social and sporting life, resulting in loss of the athlete’s identity, which, as he indicates, had a significant role in his life before the accident; the relatively short period that has passed since the SCI; and the age of the respondent making adaptation difficult. The low PILS score in R4 seems to be related to his depressive personality, both pre- and post-traumatic. A significant positive association was found between adaptation and retrospectively evaluated pre-injury personality. Specifically, patients who reported that their pre-injury personality was depressive or anxious-related also presented less adjustment [19,29]. The Kleftaras and Psarra studies showed a statistically significant negative correlation between the level of an individual’s depressive symptomatology and the total meaning of life that he feels [30]. R4 also points to the significant role of sport in shaping one’s personality, so a low PILS result may arise from a lack of this aspect in life and loss of purpose. Other participants remain connected to the sporting environment as a coach, motivator, or active participant at an amateur or professional level, which may explain the high score. Active participation in sport after an accident is also an aspect that gives meaning to life and prevents the loss of sporting identity. R3 emphasizes that sport before the accident has helped her develop perseverance and diligence. According to Kop and Jecauc, high emotional intelligence, which consists of personal competencies (self-awareness, self-regulation, and motivation) and social competencies, correlates with high sports performance [31]. It can therefore be concluded that a strong personality helps one to continue to function after SCI. This may result in greater perseverance and goal orientation, as well as greater resilience to the circumstances despite starting a changed life. Some studies revealed that individuals with strong athletic identity before the SCI can have adaptation difficulties after injury, while others showed that athletic identity has been reported as a factor that can promote recovery [32,33,34]. The results and statements of our studies support the second thesis.

Our results confirm those reported in unrepresentative samples suggesting that peri-traumatic factors (social support, pre-traumatic personality) appear to play a key role in the development and maintenance of post-traumatic responses [35,36]. Moreover, by coping with adversities such as SCIs, individuals appear to be able to experience growth, underlining the suggestion that the traumatic outcome of the disease is multidimensional, covering both negative and positive aspects [37,38,39]. In particular, this supports the thesis that self-perceived post-traumatic growth is both a coping effort and a coping effect [39].

A study by Saunders et al. indicated that increased time since injury was a protective factor in the occurrence of major depressive disorder [40]. R4 had the shortest time since SCI of all subjects. His depressive tendencies and lowest sense of meaning in life may be explained by the stage of transition from depression and despair to acceptance of the new reality and desire to grow. Another factor contributing to R4’s low scores may be his age. He is the oldest of all the subjects and shows the greatest difficulty in adjusting to the new situation. Similar findings were noted in a study by Geyh et al. involving 511 participants after SCI, where participants with higher age at injury and shorter time since injury showed lower purpose in life and more negative experiences [41]. People experiencing SCI at an older age, with depressive tendencies before the accident and in the disability acceptance phase, are a group that should receive special psychological and social support. 

During the interview, R4 becomes aware of the opportunities he has despite SCI and discovers the meaning of life with a disability. This is consistent with Frank’s observations as well as the findings of Smith and Sparkes, who highlight the importance of the storyteller’s telling his own story [18,42]. At the same time, R4 himself notes that he has limited social contacts and the fact that he participated in the research and had the opportunity to tell his story helped him to see that there is something there. Therefore, it can be said that the higher scores obtained by the other research participants can be related to good and frequent social contact and telling others about their SCI experiences, which helped their self-healing process. 

In contrast to those studied by Smith and Sparkes, the participants accept the fact of moving in a wheelchair and, in addition to the rehabilitation undertaken, focus on social engagement [14]. This attitude is also highlighted in research by Byra, who noted a positive correlation between disability acceptance and post-traumatic growth and an enhancement of mental health and social adaptation in those who were positive about disability [43,44]. Previous studies indicate that the applied rehabilitation programs and interventions are a preventive factor against reducing the sense of life in people after SCI and in other diagnostic groups [10,45]. Frankl shows how physicians can be guides in their patients’ personal search for meaning by providing care-oriented conversations engaging patients amid tragedy-ridden circumstances [6,46]. Until today, his concept has been the basis and starting point for a deeper understanding of man’s condition and people’s search for meaning in life [1]. The presented group is characterized by a high sense of meaning in life, and the analysis of factors that give them meaning may be a direction and guidance in support programs for people with SCI. Similar findings in the Thomson study, that activity and sociability are positively associated with purpose in life, can also be observed among the participants of this study [10]. The results presented here show that finding meaning in life prevents the adverse psychological effects of difficult and especially tragic situations. That is why it is so important to take into account their somatic, mental, as well as existential problems when helping people with SCI [47]. The study presented by us fits perfectly into the current existential research by exploring the meaning of life among elite athletes with SCI. It is also linked with rehabilitation psychology, where the development of a trend of exploring the positive psychological aspects of life after the occurrence of the disability is observed [28].

## 5. Conclusions

A goal in life is a powerful predictor of adaptation to SCI and this is not dictated by the severity of the injury, but rather by generating a goal in life that affects mental health [17]. A person living with a SCI who feels a lesser degree of impairment may report a greater sense of life, as well as deriving a greater value from daily activities [28]. It is we who give purpose and meaning to life, regardless of circumstances.

A strong personality in outstanding athletes is helpful to continue functioning after SCI. Staying involved in the sporting environment prevents the loss of purpose and maintains a sense of meaning at a high level. This may also serve as an example for other people with severe disabilities, encouraging them to stay engaged in social and sport activities. Both telling the story of your own illness and listening to the stories of others help the process of self-healing.

Rossini et al. indicate that gaining knowledge from people living with a SCI, in many ways, provides a microcosm by which we may better understand the resilience, adaptability, and dignity of the human spirit [28]. It is therefore important to carry out further research among SCI patients from different backgrounds to improve the knowledge of how to deal with this traumatic event, to discover the meanings that generate better mental well-being and, on this basis, to implement actions aimed to help such people adapt and improve their quality of life.

### Limitations

The relatively small number of participants in this study should be considered as a limitation. While the number of participants is appropriate in numbers for thematic analysis, it is relatively low for the generalization of the findings, which is why caution is advised. More surveys are clearly expected in larger populations, even when the populations are unrepresentative. Nevertheless, the study presents the first in-depth purpose in life study of championship-level athletes with SCI.

## Figures and Tables

**Table 1:** Purpose in Life Scale—general content.

Item	General Content	Item	General Content
1	Enthusiasm/boredom	11	Having reasons to live
2	Excitement in life/routine	12	Role in life
3	Goals and aims	13	Responsibility
4	Purposefulness/senselessness	14	Free choice
5	Variety of each day	15	Preparation for death
6	Will to live	16	Suicidal tendencies
7	Plans after retiring	17	Ability to find purpose
8	Progress in achieving goals	18	Life control
9	Excitedness/despair	19	Attitude to duties
10	Worthwhileness of life	20	Purpose in life

**Table 2:** Study respondents’ sociodemographic and health data.

Respondent	Age	Nationality	Marital Status	Profession	Age When Injured	SCI Level	Medications Used
R1	40	British	Divorced	Physical Education Teacher	27	C3/4	Painkillers, antispastic, for neurogenic disorders
R2	28	Austrian	Single	Financial Advisor	24	C6/7	For neurogenic disorders
R3	23	Polish	Informal relation	Dental technology student	18	Th11/12	None
R4	54	Asian American	Married	Chiropractor	51	C6/7	Painkillers, antispastic, antidepressants
R5	30	British	Informal relation	IT network specialist	16	Th6	Antispastic, for neurogenic disorders

**Table 3:** Participants’ score on the Purpose in Life Scale.

Respondent	Score
R1	107
R2	106
R3	131
R4	67
R5	135

## Data Availability

The datasets used and/or analyzed during this study are available from the corresponding author on reasonable request.
